# Linking governance with environmental quality: a global perspective

**DOI:** 10.1038/s41598-023-42221-y

**Published:** 2023-09-12

**Authors:** Mohammad Naim Azimi, Mohammad Mafizur Rahman, Son Nghiem

**Affiliations:** 1 School of Business, University of Southern Queensland, Toowoomba, QLD 4350 Australia; 2 College of Health and Medicine, Australian National University, Canberra, ACT 2601 Australia

**Keywords:** Ecology, Environmental economics

## Abstract

Sustainable environmental quality is a global concern, and a concrete remedy to overcome this challenge is a policy priority. Therefore, this study delves into the subject and examines the effects of governance on environmental quality in 180 countries from 1999 to 2021. To maintain comparability and precision, we first classify countries into full and income-level panels and then, innovatively, construct a composite governance index (CGI) to capture the extensive effects of governance on CO_2_ emissions. Complementing the stationarity properties of the variables, we employ the cross-sectionally augmented autoregressive distributed lags model to analyze the data. Our survey yields four key findings. First, a long-run nexus between CGI, CO_2_ emissions, and other control variables is confirmed. Second, the findings indicate that CGI is crucial to improving environmental quality by reducing CO_2_ emissions across all panels. Third, we find that while CGI maintains a similar magnitude, the size of its effects substantially varies according to the income level of the underlying countries. Fourth, the findings reveal that energy consumption, population growth rate, trade openness, and urbanization contribute to environmental degradation, while financial development and the human development index are significant in reducing CO_2_ emissions. Our findings suggest specific policy implications, summing up that one common policy is not a good fit for all environmental quality measures.

## Introduction

**Figure 1:**
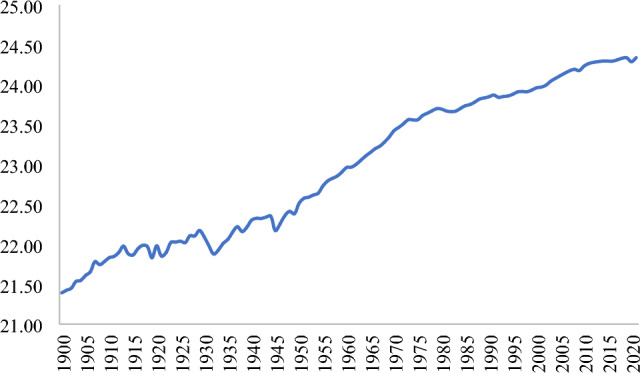
World CO_2_ emissions. Values are shown in natural logarithmic form. Source: Our World in Data.

Environmental degradation is hazardous and a global concern. The desire for a sustainabile environmental quality has increased more than ever in the contemporary period. Environmental degradation is regarded as a significant risk to achieving sustainable development goals^1^. It affects every individual, business, and society. It is a threat from which no one is immune, nor is the world able to vaccinate against it^2^. It is unanimously believed that environmental degradation caused by emitted carbon dioxide, in particular CO_2_ emissions, significantly harms humans’ lives^3^. Presently, CO_2_ emissions have crossed the determined threshold level and are sharply increasing^4^. Figure 1 shows the annual global CO_2_ emissions. It indicates that from 22.76 billion metric tons in 1900, CO_2_ emissions rose to 37.12 billion metric tons in 2020, mainly from the combustion of fossil fuels and industry. The world now emits over 34 billion metric tons per year. Evidence reveals that increased poverty, overcrowding, weather extremes, deforestation, loss of species, poor quality of water, and famine are the apparent consequences of environmental degradation. The World Bank report^5^ shows that environmental degradation caused approximately 8.1 trillion US$ damage cost in 2019, equivalent to 6.1 percent of the world’s GDP, and caused more than 90 percent of deaths in low- and middle-income countries.

Recent studies^6–10^ have identified numerous factors that can reduce the contemporary level of CO_2_ emissions. It includes controlled heating, draught-proofing, renewable energy, industrial automation with lower energy use, and many others. Undoubtedly, such subject-endogenous variables are effective in reducing CO_2_ emissions; however, the effects of exogenous factors such as good governance that might be observable in reducing emissions cannot be disregarded. Effective governance offers the necessary support for fostering a society that is essential for a better state of the environment. It is highly perceived that countries with a good governance structure are considered to have relatively better environmental quality. For example, Chaudhry et al.^11^ observed that effective institutional performance and efficient governance are substantive to promote sustainable environment. On the other hand, countries with poor governance have anemic environmental quality^12–15^. Weak social inclusion, corrupted institutions, and poor regulatory structures are found to be inimical to a sustainable environmental quality^16–18^. Leitao^19^ noticed that corruption resulting from weak governance is positively associated with CO_2_ emissions.

Prior literature has been central to warming up critical discussions on improving existing policies to enable governments to preserve environmental quality with respect to subject-endogenous factors^20–24^; however, it is important to reorient contemporary policy debates to the notion that recasting the relationships between environmental degradation and exogenous factors (say, good governance) could be a viable option^25^. Therefore, the present study primarily aims to establish the nexus between environmental degradation (CO_2_ emissions) and good governance from a global perspective; nevertheless, it would be humbler to translate the constituent objectives of the study into three research questions. First, does good governance have a long-term relationship with CO_2_ emissions? Second, what is the magnitude of the effects of good governance on CO_2_ emissions? Third, do the effects of good governance vary according to the income level of the underlying economies? Providing evidence-based answers to these questions will not only help us achieve the objectives of this investigation but will also highlight specific policy areas where good governance helps governments and policymakers reorient their existing policies.

This study is a novel piece in the existing literature from several perspectives: First, unlike recent studies that have mainly focused on the impact of governance on environmental degradation^14,17,26–28^ in regional- or country-specific contexts, the present study delves into the subject from a global perspective using a large panel of 180 countries. This approach verifies that how global emissions respond to good governance in general. Second, we innovatively construct a comprehensive composite governance index (CGI) to allow a precise evaluation of the effects of good governance on CO_2_ emissions using a distance-based approach that measures the governance from a worst-case to an ideal situation based on the data points obtained from real governance scores of the World Governance Indicators (WDI). In spite of promoting a standard measurement for good governance, this technique helps us verify the overall variability of CO_2_ emissions in the presence of major macro- and socio-economic variables. Third, to ensure capturing greater variability of environmental degradation with respect to the subject-endogenous variables, we split our panel into high-, upper-middle-, lower-middle-, and low-income countries. This approach highlights how good governance explains CO_2_ emissions across various economic statuses. Indeed, it also helps identify what specific measures policymakers should take. From a policy perspective, it is crucial to understand how the existence of good governance interplays and to what extent other socioeconomic factors influence environmental degradation. Fourth, however, in a large number of studies, it was generally assumed that good governance has an indirect impact on environmental degradation. The present study documents that good governance has the direct-influential power to explain the behavior of CO_2_ emissions across our recipient panels. Additionally, it is vital to verify that the conjecture of the direct effects of good governance can lead to the establishment of desired institutional channels to mitigate the impact of CO_2_ emissions on various social, economic, and political factors. Finally, in addition to the significant contributions of the study to the contemporary body of knowledge, the outcomes of this investigation offer specific policy implications and open a new step in the existing literature.

The remaining parts of this study are structured as follows: “Literature review” presents an extensive review of literature underpinning both theoretical and empirical issues with reference to governance-environmental nexus. “Methodology” presents the methodology, data, variables, and the econometric methods used to analyze the data. “Results and discussions” presents the statistical results. “Conclusions” concludes the study.

## Literature review

Good governance is a complex and multidimensional process of evaluating the extent to which public institutions manage the available resources, perform institutional affairs, and ensure that human rights are realized in a way that is essentially free of fraud and corruption with due consideration for the rule of law^29^. Good governance ensures that a nation’s interests are protected through effective conduits for governing and managing existing and potential resources^30^. North^31^, Greif^32^, and Acemoglu et al.^33^ promoted the concept of governance through conduits of economic, social, judicial, and political elements that highly impact macro-level policies to preserve public resources for significant social inclusion, prosperity, and the wellbeing of a nation. Theories predict that good governance plays an essential role in the formulation of policies and practices that ensure a participatory development viewpoint through increasing people’s agency in the sense of process freedom concerning environmental policies. This means allowing both governments and individuals to actively engage in, plan for, and implement policies based on their development priorities and needs^34^. Numerous studies have examined the impact of good governance on a number of socioeconomic indicators such as growth, finance, health outcomes, food insecurity, and poverty across various geographical contexts^34–37^. However, the effects of good governance on environmental degradation have not been extensively studied, but there are some studies worth reviewing. For instance, Shabir et al.^38^ investigated the effects of governance, innovative technologies, trade openness, and economic growth on CO_2_ emissions in a panel of Asia–Pacific Economic Cooperation (APEC) member countries over the period from 2004 to 2018, using the common correlated effects mean group technique. The authors observed a bidirectional link between governance and CO_2_ emissions. Wang et al.^39^ explored the asymmetric effects of institutional quality, environmental governance, and technological innovations on ecological footprints. They employed a set of panel data for European Union countries from 1990 to 2019 and a series of dynamic panel regression methods. They noticed that innovation, institutional quality, and environmental governance are crucial to reducing the ecological footprint across the reviewed countries.

Sibanda et al.^28^ examined the effects of governance on natural resources and environmental degradation from 1994 to 2020 using the generalized method of moments (GMM) technique. Their findings lend support for a statistical association between governance and environmental degradation. They also found that the rapid environmental degradation is significantly caused by the reluctance of the government to implement rules and regulations in the region. Xaisongkham and Liu^40^ delved into the effects of governance on environmental degradation in a set of selected developing economies from 2002 to 2016. The authors employed the GMM technique and found that the rule of law and government effectiveness are significant factors in reducing environmental degradation in developing countries. They suggested that sustainable environmental quality entails effective institutions to regulate human behavior with respect to environmental protection. In the same vein, Jahanger et al.^41^ used autocracy and democracy as proxies for governance quality and examined their effects on CO_2_ emissions in a panel of 69 developing countries over the period from 1990 to 2018. The authors employed panel cointegration and FMOLS methods and confirmed that governance quality has a long-run relationship with CO_2_ emissions. They also confirmed that democracy significantly reduces environmental pressures, while globalization and financial development impose adverse effects on the environment.

The literature also reveals that Azam et al.^42^ evaluated the impact of good governance on environmental quality and energy consumption in a panel of 66 developing countries for the period spanning from 1991 to 2017 using the GMM method. The authors constructed a governance index using three indicators such as political stability, administrative capacity, and democratic accountability. They observed that, though good governance has been significantly positive in affecting energy consumption, globalization has been found to be insignificant in increasing environmental quality. Moverover, Gök and Sodhi^43^ examined the link between governance and environmental quality in a panel of 115 countries classified as high-, middle-, and low-income countries from 2000 to 2015. The authors employed the system-GMM model and noticed that good governance improves environmental quality in high-income countries while having an adverse effect in middle- and low-income countries. Their conclusions suggested that improving the quality of governance is essential to environmental outcomes without tampering with existing policies. Contrary to this, Udemba^44^ investigated the effects of good governance on environmental quality in Chile using a set of time-series data from the first quarter of 1996 to the fourth quarter of 2018 and a non-linear regression approach. The author found that both good governance and foreign direct investments are statistically significant for improving environmental quality in Chile. Furthermore, Ahmed et al.^45^ examined the asymmetric effects of good governance, financial development, and trade openness on environmental degradation in Pakistan over the period from 1996 to 2018. The authors employed autoregressive distributive lags (ARDL) and non-linear ARDL models to test their hypotheses. In addition to confirming a long-run nexus between the predictors, the authors found that positive shocks to financial development and institutional quality have a significant effect on environmental degradation, while the quality of institutions is highly sensitive to enhancing environmental quality.

Akhbari and Nejati^46^ proxied governance by corruption index in a panel of 61 developing countries from 2003 to 2016 using a dynamic panel threshold model. They observed that an increase in the corruption index above a certain threshold level causes environmental quality to decrease in developing countries while having an insignificant impact below the threshold level. Dhrifi^47^ also assessed the impact of governance on environmental degradation in a panel of 45 African countries over the period 1995 to 2015 using the GMM technique. The author noticed a positive relationship between governance and environmental degradation and a negative link with health outcomes. Further, Wawrzyniak and Doryń^48^ investigated the influence of good governance on moderating the relationships between economic growth and CO_2_ emissions in a panel of 93 emerging and developing economies from 1995 to 2014. The authors used government effectiveness and control of corruption indicators as proxies for governance and employed the GMM model. Their findings revealed that government effectiveness is significant in moderating the influence of economic growth on CO2 emissions. Similarly, Samimi et al.^49^ employed a set of annually aggregated datasets for a panel of 21 countries in the Middle East and North Africa from 2002 to 2007 to examine the impact of good governance on environmental degradation. The authors used three indicators, such as government effectiveness, regulatory quality, and control of corruption, as proxies for good governance. They found that government effectiveness has a positive effect on environmental quality, while the remaining two indicators were found to be insignificant. Finally, Tamazian and Rao^50^ investigated the relationships between financial development, environmental degradation, and good governance in a panel of 24 transitional economies from 1993 to 2004. Using the standard reduced-form modeling approach and GMM models, the authors found that both financial development and good governance (institutional quality) are crucial factors for environmental performance.

Recent studies have significantly contributed to enhancing the contemporary body of knowledge in the field; however, a critical review of the cited studies reveals several gaps. First, good governance is a multifaceted concept, and its precise effects may not be well examined by using single or inconclusive proxies. For example, various studies employed different proxies for good governance, among which government effectiveness and control of corruption are the most common ones. To rectify this issue, we developed the following hypothesis:

Hypothesis 1: Composite governance index (CGI) is an accurate predictor that allows more precise evaluation of the effects of good governance on the subject.

Second, prior studies achieved conflicting results about the effects of good governance on environmental quality, leaving the subject unattended to offer specific policy implications. Therefore, to address this empirical shortcoming, the following hypothesis is developed:

Hypothesis 2: CGI has a long-term and positive link with CO_2_ emissions.

Third, the review of recent studies reveal that holistic measures to highlight global perspectives and precise comparability of the effects of good governance on environmental quality are missing. To address this empirical shortcoming, we developed the following hypothesis:

Hypothesis 3: Based on the size of the underlying economies, the effect size of good governance varies and thus exhibits non-monotonic behavior.

## Methodology

**Figure 2:**
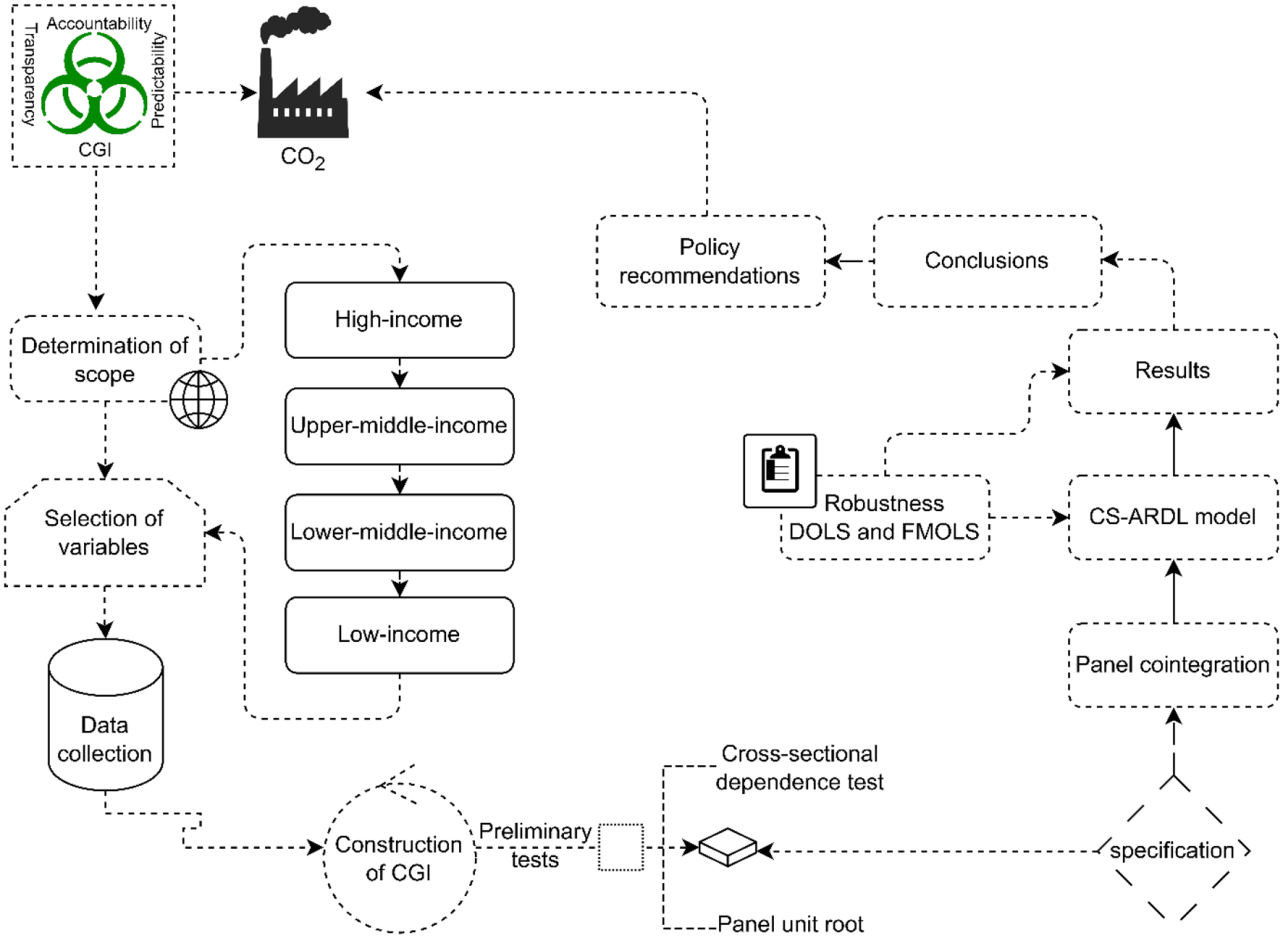
Visual abstract. Source: Authors’ creation.

In this section, we explain the methodological approach used in the study to assess the effects of good governance on CO_2_ emissions. This approach has been widely used in prior literature and leads to a systematic way of testing the hypotheses developed^51,52^. Although we describe the methods sequentially in the following sub-sections, we summarize them through a visual abstract depicted in Fig. 2.

### Data presentation

The present study focuses on the effects of good governance on environmental degradation in 180 countries from 1999 to the most recent updated datasets in 2021. Table 1 presents the list of reviewed countries. Based on the primary objective of the study, we first group the countries into a full panel and then into income level categories such as high-income (HIC), upper-middle-income (UMIC), lower-middle-income (LMIC), and low-income (LIC). The classification is based on the World Bank’s^53^ report and allows us to maintain rational comparability of the results to offer a global image of the nexus between good governance and environmental degradation.

**Table 1:** List of countries.

No	Country	Income	No	Country	Income	No	Country	Income	No	Country	Income
1	Andorra	HIC	46	Panama	HIC	91	South Sudan	LIC	136	Argentina	UMIC
2	Antigua and Barbuda	HIC	47	Poland	HIC	92	Sudan	LIC	137	Armenia	UMIC
3	Aruba	HIC	48	Portugal	HIC	93	Syrian Arab Republic	LIC	138	Azerbaijan	UMIC
4	Australia	HIC	49	Puerto Rico	HIC	94	Tajikistan	LIC	139	Belarus	UMIC
5	Austria	HIC	50	Qatar	HIC	95	Togo	LIC	140	Belize	UMIC
6	Bahamas	HIC	51	Romania	HIC	96	Uganda	LIC	141	Bosnia and Herzegovina	UMIC
7	Bahrain	HIC	52	San Marino	HIC	97	Yemen, Rep	LIC	142	Botswana	UMIC
8	Barbados	HIC	53	Saudi Arabia	HIC	98	Algeria	LMIC	143	Brazil	UMIC
9	Belgium	HIC	54	Seychelles	HIC	99	Angola	LMIC	144	Bulgaria	UMIC
10	Bermuda	HIC	55	Sint Maarten (Dutch part)	HIC	100	Bangladesh	LMIC	145	Colombia	UMIC
11	British Virgin Islands	HIC	56	Slovak Republic	HIC	101	Benin	LMIC	146	Costa Rica	UMIC
12	Canada	HIC	57	Slovenia	HIC	102	Bhutan	LMIC	147	Cuba	UMIC
13	Cayman Islands	HIC	58	Spain	HIC	103	Bolivia	LMIC	148	Dominica	UMIC
14	Channel Islands	HIC	59	St. Kitts and Nevis	HIC	104	Cabo Verde	LMIC	149	Dominican Rep	UMIC
15	Chile	HIC	60	St. Martin (French part)	HIC	105	Cameroon	LMIC	150	Ecuador	UMIC
16	Croatia	HIC	61	Sweden	HIC	106	Comoros	LMIC	151	Equatorial Guinea	UMIC
17	Curaçao	HIC	62	Switzerland	HIC	107	Congo, Rep	LMIC	152	Gabon	UMIC
18	Cyprus	HIC	63	Trinidad and Tobago	HIC	108	Côte d'Ivoire	LMIC	153	Georgia	UMIC
19	Czech Republic	HIC	64	Turks and Caicos Islands	HIC	109	Djibouti	LMIC	154	Grenada	UMIC
20	Denmark	HIC	65	United Arab Emirates	HIC	110	Egypt, Arab Rep	LMIC	155	Guatemala	UMIC
21	Estonia	HIC	66	United Kingdom	HIC	111	El Salvador	LMIC	156	Guyana	UMIC
22	Faroe Islands	HIC	67	United States	HIC	112	Eswatini	LMIC	157	Iran, Islamic Rep	UMIC
23	Finland	HIC	68	Uruguay	HIC	113	Ghana	LMIC	158	Iraq	UMIC
24	France	HIC	69	Virgin Islands (U.S.)	HIC	114	Honduras	LMIC	159	Jamaica	UMIC
25	Germany	HIC	70	Afghanistan	LIC	115	India	LMIC	160	Jordan	UMIC
26	Gibraltar	HIC	71	Burkina Faso	LIC	116	Kenya	LMIC	161	Kazakhstan	UMIC
27	Greece	HIC	72	Burundi	LIC	117	Kyrgyz Republic	LMIC	162	Kosovo	UMIC
28	Greenland	HIC	73	Central African Republic	LIC	118	Lesotho	LMIC	163	Lebanon	UMIC
29	Hungary	HIC	74	Chad	LIC	119	Mauritania	LMIC	164	Libya	UMIC
30	Iceland	HIC	75	Congo, Dem. Rep	LIC	120	Moldova	LMIC	165	Maldives	UMIC
31	Ireland	HIC	76	Eritrea	LIC	121	Morocco	LMIC	166	Mexico	UMIC
32	Isle of Man	HIC	77	Ethiopia	LIC	122	Nepal	LMIC	167	Montenegro	UMIC
33	Israel	HIC	78	Gambia	LIC	123	Nicaragua	LMIC	168	Namibia	UMIC
34	Italy	HIC	79	Guinea	LIC	124	Nigeria	LMIC	169	North Macedonia	UMIC
35	Kuwait	HIC	80	Guinea-Bissau	LIC	125	Pakistan	LMIC	170	Paraguay	UMIC
36	Latvia	HIC	81	Haiti	LIC	126	São Tomé and Principe	LMIC	171	Peru	UMIC
37	Liechtenstein	HIC	82	Liberia	LIC	127	Senegal	LMIC	172	Russian Federation	UMIC
38	Lithuania	HIC	83	Madagascar	LIC	128	Sri Lanka	LMIC	173	Serbia	UMIC
39	Luxembourg	HIC	84	Malawi	LIC	129	Tanzania	LMIC	174	South Africa	UMIC
40	Malta	HIC	85	Mali	LIC	130	Tunisia	LMIC	175	St. Lucia	UMIC
41	Mauritius	HIC	86	Mozambique	LIC	131	Ukraine	LMIC	176	St. Vincent and the Grenadines	UMIC
42	Monaco	HIC	87	Niger	LIC	132	Uzbekistan	LMIC	177	Suriname	UMIC
43	Netherlands	HIC	88	Rwanda	LIC	133	Zambia	LMIC	178	Turkey	UMIC
44	Norway	HIC	89	Sierra Leone	LIC	134	Zimbabwe	LMIC	179	Turkmenistan	UMIC
45	Oman	HIC	90	Somalia	LIC	135	Albania	UMIC	180	Venezuela, RB	UMIC

### Selection and description of variables

We use a set of variables that are consistent with the theoretical framework and recent empirical works (see, for instance,^54–56^), except for the CGI, which is innovatively constructed to capture the extensive effects of good governance on the subject. The variables are described as follows:

#### Measurement of environmental quality

CO_2_ emissions (CO_2_) have been used as the dependent variable. It is expressed in metric tons per capita. CO_2_ stems from the combustion of fossil fuels and the manufacture of cement. It includes carbon dioxide produced during the consumption of solid, liquid, and gas fuels and gas flaring^57^.

#### Measurement of good governance

A comprehensive composite governance index (CGI) has been constructed using the proposed methodology by Sarma^58^ and six governance indicators such as control of corruption, government effectiveness, political stability, the rule of law, regulatory quality, and voice and accountability. For two reasons, it is important to construct a CGI. First, it is a more efficient approach to exploring the extensive effects of good governance on the subject compared to individual indicators and other index construction methods. Second, the incorporation of CGI allows the study to include more control predictors, leading to an appropriate specification and more accurate results^59–61^. Table A1 of Appendix A explains CGI’s construction process in detail. CGI is expressed in numbers ranging from 0 (imperfect) to 1 (perfect) governance.

#### Measurement of income level

GDP growth rate (EG) has been used to present economic variations through various stages of development at which CO_2_ emissions are produced^62^. EG is expressed as an annual percentage.

#### Measurement of financial development

The financial development index (FDI) of the International Monetary Fund has been used as the best-fit proxy for financial development. FDI is expressed in numbers from 0 to 1 (high). Recent studies indicate that financial development influences CO_2_ emissions^63,64^. Therefore, we control for the effects of FDI on CO_2_ emissions.

#### Measurement of energy consumption

Energy consumption (EGY), expressed in kilograms of oil equivalent per capita, is used as a control variable. Recent studies suggest the use of EGY as a key pollutant predictor in the analysis of environmental quality and other socioeconomic indicators. It is evident that EGY supports higher growth^65^, while it also increases the use of fossil fuels, resulting in higher CO_2_ emissions. Chontanawat^66^ and Elfaki et al.^67^ argue that there is a triangle causal link between EGY, EG, and CO_2_ emissions.

#### Measurement of human interaction

In order to control for the effects of human interaction on CO2 emissions, we employ three common variables, namely, the human development index (HDI), population growth rate (PGR), and urbanization (URB). HDI, PGR, and URB, respectively, are expressed in numbers from 0 to 1 (high), annual growth rate, and percentage of the total population. Studies indicate that human intervention has substantively disturbed the contemporary ecosystem. However, effective administration of human activities, as well as utilizing their potential, may improve environmental quality^21,68^. Moreover, a higher proportion of greenhouse gas emissions is linked to the process of global urbanization, which is primarily evident in nations following growth-targeting regimes^56^. These emissions are mostly produced by construction projects, higher energy consumption, and the use of chemical materials.

#### Measurement of trade openness

Trade openness (TOP), expressed as a percentage of GDP, is our final control variable. Though recent literature is largely inconclusive about the effects of TOP on CO_2_ emissions^69^, two main findings—positive and negative impacts—are evident. The study incorporates TOP into the analysis to avoid any potential spuriousness.

#### Sources of data

The datasets relevant to governance indicators come from Worldwide Governance Indicators (WGI). The datasets for FDI have been collected from the International Monetary Fund (IMF), while the required datasets for HDI were compiled from PWT 9.0 (Penn World Table), sourced from Feenstra et al.^70^. The data for all other variables has been collected from World Development Indicators (WDI).

### Model specification

Our main primary objective is to examine the effects of CGI—that is, the composite governance index—on CO_2_ emissions in a large panel to represent a global image. Assuming that good governance is essential to environmental quality, as suggested by theoretical expectations of institutional impacts^71^, we initiate with the following dynamic panel multivariate specification:

1 $$\begin{gathered} CO_{2it} = \eta_{i} + \lambda_{1i} CGI_{it} + \lambda_{2i} EG_{it} + \lambda_{3i} EGY_{it} + \lambda_{4i} FDI_{it} + \lambda_{5i} PGR_{it} \hfill \\ \,\,\,\,\,\,\,\,\,\,\,\,\, + \lambda_{6i} TOP_{it} + \lambda_{7i} HDI_{it} + \lambda_{8i} URB_{it} + n_{t} + u_{it} \hfill \\ \end{gathered}$$

where all variables are defined before, $\eta_{i} =$ intercept, $\lambda_{1i} ,...,\lambda_{8i} =$ long-run coefficients, and $n_{t} =$ country-specific unobserved effects. The estimation of Eq. (1) requires us to select and compute a number of econometric techniques that are explained in the following sub-sections.

### Cross-sectional dependence test

In panel data analysis, appropriate specification requires several prior estimations, one of which, in particular, is the cross-sectional dependence (CD) test. Rapid globalization, unrestricted trade, common technological deployment, and capital mobility are some obvious reasons why countries may exhibit CD^72^. Thus, we begin with the CD test of Pesaran^73^, which takes the following form:

2 $$CD = \sqrt {\frac{2T}{{N(N - 1)}}} \left( {\sum\nolimits_{i = 1}^{N - 1} {\sum\nolimits_{j = i + 1}^{N} {\sqrt {T_{ij} \overset{\lower0.5em\hbox{$\smash{\scriptscriptstyle\frown}$}}{\rho }_{ij} } } } } \right)$$

where $$\overset{\lower0.5em\hbox{$\smash{\scriptscriptstyle\frown}$}}{\rho }_{ij}$$ is the sample estimates of the pair-wise correlation of the residuals and “$$T_{ij} = \# \,\,(T_{i} \cap T_{j} )$$ is the number of common time-series observations between unit $$i$$ and $$j.$$” Eq. (2) shows that under the null hypothesis of no cross-sectional dependence $$CD\,\,^{\underrightarrow d} \,\,N(0,1)\,\,{\text{for}}\,\,N \to \infty \,\,{\text{and}}\,\,T$$^74^. To ensure the robustness of the results obtained from Eq. (2), we use the proposed model of Pesaran and Yamagata^75^ to tes the null of slope homogeneity of the panels under review.

### Stationarity test

Next, in light of the rejected null of no CD, the common panel unit root test may generate inconsistent results that may lead to misspecification. Therefore, we use the proposed test of Pesaran^76^, the so-called CIPS (cross-sectionally augmented Im, Pesaran, and Shin) method. It is based on the foundational cross-sectionally augmented Dickey and Fuller (CADF) test with augmented cross-sectional mean $\overline{y}_{it}$ and differenced cross-sectional mean value $\Delta \overline{y}_{it}$ of the variables under review as follows:

3 $$\Delta y_{it} = \eta_{i} + \gamma_{1i} y_{i,t - 1} + \gamma_{2i} \overline{y}_{i,t - 1} + \vartheta_{i} \Delta \overline{y}_{t} + u_{it}$$

where as a common test of the null $\gamma = 0$ for every $i$ against its alternative $\gamma_{i} < 0,...,\gamma_{N0} < 0,N_{0} \le N$ and then, given by the average of individual CADF as:

4 $$CIPS(N,T) = N^{ - 1} \sum\nolimits_{i = 1}^{N} {CADF_{i} }$$

As a common practice, it notes that for the rejected null of panel non-stationarity, the critical value of a desired significant level must be less than the CIPS test statistics at the level. CIPS is advantageous over other panel unit root tests. It neatly detects the true stationarity of the panel variables arising from common unobserved factors^76^, thus leading to an appropriate specification.

### Cointegration tests

Again, for the rejected null of no CD, common panel cointegration techniques may be biased. Thus, we employ the proposed model of the Westerlund^77^ test, which has two key advantages over other panel cointegration methods. First, it accounts for the effects of any CD existing in the panel, and second, it considers the lead-lag length for small samples. The study employs the following compact form of the test:

5 $$\begin{gathered} \Delta y_{it} = \eta^{\prime}_{i} d_{t} + \varphi_{i} \left( {y_{it - 1} - \theta^{\prime}_{i} x_{it - 1} } \right) \hfill \\ \,\,\,\,\,\,\,\,\,\, + \sum\nolimits_{j = 1}^{{p_{i} }} {\varphi_{ij} \Delta y_{it - j} } + \sum\nolimits_{{j = q_{i} }}^{{p_{i} }} {\vartheta_{ij} \Delta x_{it - j} + \varepsilon_{it} } \hfill \\ \end{gathered}$$

where $d_{t} (1,t)^{\prime} =$ deterministic regressor, $\eta^{\prime}_{i} =$ vector of parameters $(\eta_{1i} ,\eta_{2i} )^{\prime},$ and other parameters hold similar meaning as explained before. To estimate the error-corrected form through the least squares method, we modeify Eq. (5) and represent it as follows:

6 $$\begin{gathered} \Delta y_{it} = \eta^{\prime}_{i} d_{t} + \varphi_{i} y_{it - 1} + \theta^{\prime}_{i} x_{it - 1} \hfill \\ \,\,\,\,\,\,\,\,\,\, + \sum\nolimits_{j = 1}^{{p_{i} }} {\varphi_{ij} \Delta y_{it - j} } + \sum\nolimits_{{j = - q_{i} }}^{{p_{i} }} {\vartheta_{ij} \Delta x_{it - j} + \varepsilon_{it} } , \hfill \\ \,\,\,\,\theta^{\prime}_{i} = - \varphi_{i} \beta^{\prime}_{i} \hfill \\ \end{gathered}$$

Having all vectors defined before,$\varphi_{i} =$ speed of adjustment at which the model returns to its initial equilibrium. Moreover, Eq. (6) adjusts the errors to be independent across all $t$ and $i.$ It also corrects for any CD through bootstrapping method.

### CS-ARDL model

To examine the effects of CGI and other control variables on CO_2_ emissions in a group of panels, we use the CS-ARDL (cross-sectionally augmented autoregressive distributed lags) model of Chudik and Pesaran^78^, which is an appropriate technique for the case of our inquiry. The rationality of using the CS-ARDL model is based on two key empirical reasons. First, for the rejected null of no CD, common panel techniques fail to capture the true effects and may produce inconsistent and biased coefficients. Second, it corrects any slope heterogeneity and allows the variables to exhibit mixed stationarity properties. Having said that, we proceed to specify the CS-ARDL model by augmenting the symmetric ARDL with a linear combination of cross-sectional mean values of the lagged dependent variable and explanatory variables to capture the CD in the error term as follows:

7 $$\begin{gathered} \Delta y_{it} = \vartheta_{i} + \lambda_{i} \left( {y_{it - 1} - \eta_{i}{\prime} x_{it - 1} + \varphi_{i}^{ - 1} \overline{y}_{t} + \varphi_{i}^{ - 1} \xi ^{\prime}\overline{x}_{t} } \right) + \sum\nolimits_{j = 0}^{u - 1} {\gamma_{ij} \Delta y_{it - j} } \, \hfill \\ \,\,\,\,\,\,\,\,\,\, + \sum\nolimits_{j = 0}^{v - 1} {\phi_{ij} \Delta x_{it - j} } + \sum\nolimits_{j = 0}^{u - 1} {\xi_{ik} \Delta \overline{y}_{t - j} } + \sum\nolimits_{j = 0}^{v - 1} {\varpi_{ij} \Delta \overline{x}_{t - j} } + u_{it} \hfill \\ \end{gathered}$$

where $y_{it} =$ dependent variable, $x_{it} =$ explanatory variables, $u =$ lag operator of the dependent variable, $v =$ lag operator of the independent variables, $\vartheta_{i} =$ intercept, $\lambda_{i} =$ speed of adjustment of panel to its long-run equilibrium, $(y_{it - 1} - \eta_{i}{\prime} x_{it - 1} + \varphi_{i}^{ - 1} \overline{y}_{t} + \varphi_{i}^{ - 1} \xi ^{\prime}\overline{x}_{t} ) =$ level terms of CD and cointegration between variables, $\overline{y} =$ cross-sectional mean of the dependent variable, $\overline{x} =$ cross-sectional mean of the explanatory variables, $\lambda_{i} ,\,\,\eta_{i} ,\,\,{\text{and}}\,\,\varphi_{i} =$ long-run coefficients, $\gamma_{i} ,\,\,\phi_{i,} \,\,\xi_{i} ,\,\,{\text{and}}\,\,\varpi_{i} =$ short-run coefficients, and $u_{it} =$ the error-term. Equation (6) uses either mean group or pooled mean group estimators, the choice of which depends on the homogeneity of the slope coefficients of the long-term effects^79^. In our dynamic panel estimations, the study employs the pooled mean group estimator, observing it as an appropriate method to preserve the consistency and efficiency of coefficients.

### FMOLS and DOLS tests

As suggested by prior literature (refer^38,80,81^), we test the robustness of our estimated outcomes obtained from the computation of Eq. (7) using the fully modified least squares (FMOLS) and dynamic ordinary least squares (DOLS) techniques. Phillips and Hansen^82^ developed the FMOLS model to estimate an optimal cointegrating equation; however, based on the preference for correcting serial correlation and endogeneity bias, we apply the FMOLS method proposed by Pedroni^83^ as expressed below:

8 $$\begin{gathered} \beta_{NT}^{*} - \beta = \left( {\sum\nolimits_{i = 1}^{N} {L_{22i}^{ - 2} } \sum\nolimits_{i = 1}^{T} {\left( {y_{it} - \overline{y}_{it} } \right)^{2} } } \right)\sum\nolimits_{i = 1}^{N} {L_{11i}^{ - 1} } L_{22i}^{ - 1} \hfill \\ \,\,\,\,\,\,\,\,\,\,\,\,\,\,\,\,\,\,\,\,\,\,\,\left( {\sum\nolimits_{i = 1}^{T} {\left( {y_{it} - \overline{y}i} \right)\eta_{it}^{*} } - T\overset{\lower0.5em\hbox{$\smash{\scriptscriptstyle\frown}$}}{\psi }_{i} } \right)\,\,\, \hfill \\ \end{gathered}$$

where $$\eta_{it}^{*} = \eta_{it} - (\overset{\lower0.5em\hbox{$\smash{\scriptscriptstyle\frown}$}}{L}_{21i} /\overset{\lower0.5em\hbox{$\smash{\scriptscriptstyle\frown}$}}{L}_{22i} )\Delta y_{it} ,\,\,\,\overset{\lower0.5em\hbox{$\smash{\scriptscriptstyle\frown}$}}{\psi }_{i} = \overset{\lower0.5em\hbox{$\smash{\scriptscriptstyle\frown}$}}{\zeta }_{21i} \overset{\lower0.5em\hbox{$\smash{\scriptscriptstyle\frown}$}}{\Xi }_{{_{21i} }}^{0} - (\overset{\lower0.5em\hbox{$\smash{\scriptscriptstyle\frown}$}}{L}_{21i} /\overset{\lower0.5em\hbox{$\smash{\scriptscriptstyle\frown}$}}{L}_{22i} )(\overset{\lower0.5em\hbox{$\smash{\scriptscriptstyle\frown}$}}{\zeta }_{22i} + \overset{\lower0.5em\hbox{$\smash{\scriptscriptstyle\frown}$}}{\Xi }_{{_{22i} }}^{0} )$$ and $$\overset{\lower0.5em\hbox{$\smash{\scriptscriptstyle\frown}$}}{L}_{i}$$ presents the lower triangulation of $$\overset{\lower0.5em\hbox{$\smash{\scriptscriptstyle\frown}$}}{\Xi }_{i} .$$ Moreover, DOLS is a rather parametric technique and has a similar asymptotic distribution to that of FMOLS^84^. We use and report both to confirm the consistency and robustness of the estimated outcomes.

## Results and discussions

### Descriptive summary

A summary of statistics has been provided to reflect the overall state of the predictors used in the study. While Table 2 presents the summary statistics for the full panel, it also disaggregates them by income-level groups. Though one can read through it, it shows that the mean values of CO_2_ emissions and CGI, two variables of interest, are 5.091 metric tons per capita and 0.746, respectively, in the full panel. Better insight is achieved when the statistics are compared by income level. It shows that despite the fact that HIC has the highest mean value of the CGI, it also produces the highest CO_2_ emissions compared to UMIC, LMIC, and LIMC.

**Table 2:** Summary statistics.

Variables	CO_2_	CGI	EGY	EG	FDI	HDI	PGR	TOP	URB
Full panel									
Mean	5.091	0.746	9.224	3.366	0.382	0.667	1.340	87.061	58.367
Standard deviation	6.648	0.293	1.728	5.441	0.240	0.195	1.719	50.450	23.886
Mininum	0.019	0.026	0.567	−50.339	0.039	0.017	−18.530	11.093	4.017
Maximum	62.259	0.987	13.396	86.827	0.975	0.962	21.260	468.568	100.00
Observations	4140	4140	4140	4140	4140	4140	4140	4140	4140
HIC									
Mean	8.614	0.796	9.494	2.563	0.486	0.794	1.187	100.09	73.586
Standard deviation	8.431	0.319	1.767	4.400	0.234	0.170	1.743	54.679	20.674
Mininum	0.136	0.045	3.180	−23.508	0.026	0.110	−11.99	38.680	14.303
Maximum	11.259	0.847	13.396	33.629	0.975	0.962	21.260	408.362	69.853
Observations	1587	1587	1587	1587	1587	1587	1587	1587	1587
UMIC									
Mean	3.943	0.778	3.729	9.398	0.213	0.970	1.093	78.033	61.356
Standard deviation	3.443	0.327	1.590	6.424	0.409	0.168	1.521	35.390	9.709
Mininum	5.067	0.347	1.664	17.099	0.110	0.015	−9.061	14.382	14.317
Maximum	18.437	0.794	12.908	63.480	0.739	0.805	12.00	236.00	92.17
Observations	1059	1059	1059	1059	1059	1059	1059	1059	1059
LMIC									
Mean	2.482	0.726	9.058	3.885	0.332	0.564	1.709	89.779	46.128
Standard deviation	0.135	0.258	1.620	0.231	0.227	0.127	1.925	60.846	16.337
Mininum	0.093	0.244	4.180	−29.100	0.019	0.189	−4.950	22.619	13.379
Maximum	31.105	0.886	11.983	21.452	0.917	0.786	18.910	468.568	78.380
Observations	851	851	851	851	851	851	851	851	851
LIC									
Mean	1.721	0.651	8.470	4.031	0.292	0.437	1.616	66.196	32.077
Standard deviation	3.745	0.290	1.787	0.974	0.240	0.124	1.625	32.517	12.279
Mininum	0.019	0.022	4.596	−50.339	0.030	0.019	−18.53	1.862	8.246
Maximum	21.996	0.807	11.431	86.827	0.910	0.802	9.900	290.499	63.852
Observations	643	643	643	643	643	643	643	643	643

**Figure 3:**
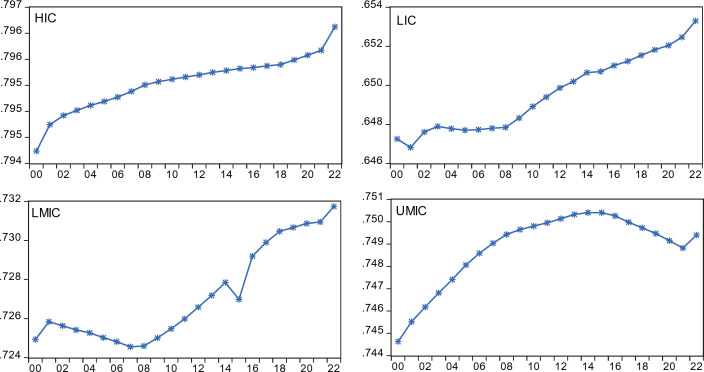
CGI annual average plot by income group.

**Figure 4:**
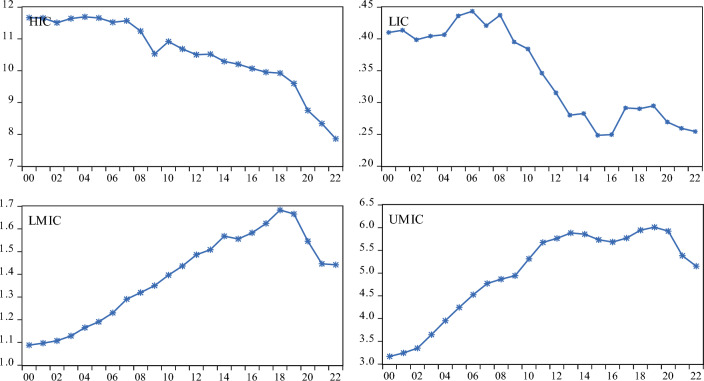
Annual CO_2_ emissions plot.

Using actual series, we further generate an annual average trend of the CGI and CO_2_ emissions across income groups and depict them in Figs. 3 and 4, respectively.

Figure 3 indicates that CGI has been smoothly improving over the years in HIC and LIC, while LMIC and UMIC panels exhibit some structural breaks. On the other hand, CO_2_ emissions were significantly reduced over the years in HIC and LIC, whereas in UMIC and LMIC, a downward shift was only evident from 2018 onwards (Fig. 4). Furthermore, before any empirical analysis, we estimated the pairwise correlation matrix and found that there is no significant correlation between all the variables, both in the full and income-level groups. To save space, we avoided reporting the correlation analysis, but it can be provided upon request.

### CD and unit root test results

Prior to any inferences, as described earlier, the study tests the null of no CD among the full and the income-level panels. The results are reported in Table 3 and indicate that except for URB in full, UMIC, and LMIC panels, all other variables are significant to reject the null of no CD. Moreover, the study examines the slope heterogeneity using the proposed model of Pesaran and Yamagata^85^. It is also used to ensure that the results derived from the CD test are consistent. The results reported in Tables 4 and 5 indicate that for all panels, the null is rejected at 1% and 5% significant levels, implying that the slopes are heterogeneous across the panels.

**Table 3:** CD test results.

Variables	Full panel		HIC		UMIC		LMIC		LIC	
	CD-test	p-value	CD-test	p-value	CD-test	p-value	CD-test	p-value	CD-test	p-value
CO_2_	33.44***	0.000	41.16***	0.000	38.12***	0.000	44.42***	0.000	24.87***	0.000
CGI	28.16***	0.000	15.18***	0.000	40.01***	0.000	15.06***	0.000	12.22***	0.000
EGY	11.79***	0.000	1.86*	0.063	11.09***	0.000	23.53***	0.000	6.41***	0.000
EG	160.04***	0.000	92.49***	0.000	64.44***	0.000	2.32**	0.020	2.39**	0.026
FDI	96.31***	0.000	46.51***	0.000	30.98***	0.000	30.08***	0.000	7.55***	0.000
HDI	41.06***	0.000	174.56***	0.000	85.13***	0.000	16.98***	0.000	6.66***	0.000
PGR	79.20***	0.000	31.02***	0.000	29.76***	0.000	93.16***	0.000	40.65	0.000
TOP	62.30***	0.000	41.00***	0.000	36.55***	0.000	15.91***	0.000	6.23***	0.000
URB	1.09	0.225	0.95	0.410	1.15	0.220	86.09***	0.000	84.63***	0.000

***, **, and * indicate significance at 1%, 5%, and 10%, respectively.

**Table 4:** Slope heterogeniety test results.

Variables	Full panel		HIC		UMIC		LMIC		LIC	
	Delta	p-value	Delta	p-value	Delta	p-value	Delta	p-value	Delta	p-value
Unadjusted	20.047***	0.000	12.948***	0.000	9.473***	0.000	0.508	0.611	−0.648	0.517
Adjusted	26.665***	0.000	17.222***	0.000	13.081***	0.000	0.676	0.499	−0.862	0.389

***, **, and * indicate significance at 1%, 5%, and 10%, respectively.

**Table 5:** CIPS unit root test results.

Variables	Full panel		HIC		UMIC		LMIC		LIC	
	I(0)	I(1)	I(0)	I(1)	I(0)	I(1)	I(0)	I(1)	I(0)	I(1)
CO_2_	−1.52	−4.65***	−1.74	−4.71***	−1.44	−4.18***	−2.21**	−4.63***	−1.08	−3.86***
CGI	−1.69	−3.52***	−1.57	−3.48***	−1.26	−3.99***	−1.79	−4.08***	−1.26	−4.37***
EGY	−1.54	−4.44***	−1.56	−4.56***	−1.38	−4.13***	−1.86	−4.79***	−0.84	−3.65***
EG	−3.57***	−5.45***	−3.65***	−5.31***	−0.89	−3.97***	−3.24***	−5.56***	−1.33	−4.17***
FDI	−2.28***	−4.49***	−2.00	−4.49***	−3.47***	−5.09***	−2.49***	−4.98***	−4.05***	−6.02***
HDI	−2.05*	−3.60***	−1.92	−3.99***	−3.54***	−5.12***	−2.22**	−2.75***	−0.99	−3.67***
PGR	−1.80	−3.67***	−1.75	−3.75***	−1.04	−3.99***	−1.31	3.70***	−1.45	−4.29***
TOP	−1.60	−4.21***	−1.63	−4.02***	−1.62	−4.22***	−1.81	−4.49***	−3.59***	−4.78***
URB	−0.75	−3.26***	−0.81	−2.21***	−1.18	−4.15***	−1.17	−4.11***	−1.11	−3.79***

***, **, and * indicate significance at 1%, 5%, and 10%, respectively.

The results obtained from the CD test suggest examining the unit root of the variables. To this end, we use the CIPS test of Pesaran^76^ and report the results in Table 7. The results demonstrate that EG, FDI, and HDI are significant to reject the null of the unit root at the level, while the remaining variables are first-difference stationary in the full and LMIC panels. For HIC, only EG is level-stationary, and the other predictors are first-differenced stationary. The results for the UMIC panel, FDI, and HDI reject the null, while others become significant to reject the null after the first difference. Finally, LIC also shows mixed integration. It indicates that FDI and TOP can reject the null, while other variables display first-differenced stationarity.

### Panel cointegration results

In the presence of CD and slope heterogeneity across the full and income-level panels, the study computes Westerlund’s^77^ panel cointegration test. The results are presented in Table 6. The findings demonstrate that, with no exception, there exists a long-run relationship between the variables. For instance, for the full panel, the variance ratio is 3.96 with a corresponding p-value of < 0.01, which significantly rejects the null of no panel cointegration. However, the variance ratio for HIC is significant at 5% to reject the null, and other income-level panels are significant at a 1% level.

**Table 6:** Westerlund cointegration test results.

Estimated model	Full panel		HIC		UMIC		LMIC		LIC	
	Statistics	p-value	Statistics	p-value	Statistics	p-value	Statistics	p-value	Statistics	p-value
Variance ratio	3.96***	0.000	1.81**	0.034	4.57***	0.000	2.62***	0.004	3.019***	0.001
Countries (N)	180		69		47		37		28	
Time period (T)	23		23		23		23		23	
Observations	4140		1578		1059		851		643	

***, **, and * indicate significance at 1%, 5%, and 10%, respectively.

The findings are consistent with theoretical and empirical expectations, and they support the notion that in the long run, CO_2_ emissions move in tandem with CGI and other control variables augmented in the study. Additionally, our results are consistent with the findings of Goel et al.^86^, Lau et al.^87^, and Fatima et al.^88^, who also documented significant cointegrations between the predictors of institutional quality and CO_2_ emissions. While cointegrated vectors suggest that CGI and other control variables affect CO_2_ emissions differently across all panels, they also trigger delving into the scale and magnitude of the effects of CGI and control variables on the subject matter. Thus, we proceed to estimate the CS-ARDL model in the following section.

### CS-ARDL estimates

All prior estimations (Tables 2, 3, 4, 5 and 6), and the cointegration results in particular, have been conducive to one of the objectives of this inquiry. In Table 7, we present the main empirical findings of the study obtained through the estimation of the CS-ARDL model expressed in Eq. (7). Table 7 simplifies the results. Its upper part reports the short-run estimates, while the lower part presents the long-run effects. Diagnostic checks are offered underneath long-run estimation, and the row-ordered panels of the table offer an inter-group comparative analysis.

**Table 7:** CS-ARDL test results.

	(1) Full panel		(2) HIC		(3) UMIC		(4) LMIC		(5) LIC	
Variables	Coefficient	p-value	Coefficient	p-value	Coefficient	p-value	Coefficient	p-value	Coefficient	p-value
Short-run effects										
ΔCO_2*t*−1_	0.728***	0.000	0.917***	0.000	0.699***	0.005	0.826***	0.000	−0.868***	0.000
ΔCGI	−0.179	0.225	−0.565	0.186	−0.198	0.410	−0.069	0.735	0.150	0.337
ΔEGY	0.328*	0.075	0.717***	0.004	0.522***	0.000	0.191***	0.000	0.229***	0.000
ΔEG	0.021***	0.001	−0.035	0.260	0.047*	0.081	0.061*	0.087	0.023***	0.003
ΔFDI	−0.172***	0.000	−0.602***	0.000	−0.508	0.420	−0.966	0.413	−0.981	0.279
ΔHDI	−0.746*	0.056	−0.248***	0.008	-0.194	0.575	−0.211	0.899	-0.558	0.620
ΔPGR	0.359***	0.000	0.460***	0.000	0.355	0.422	0.279	0.575	0.318	0.555
ΔTOP	0.043	0.124	0.018***	0.000	0.037	0.845	0.033	0.310	0.275	0.320
ΔURB	0.462***	0.000	0.977	0.384	0.251	0.145	0.399	0.286	0.147**	0.031
*ECT*	−0.992***	0.000	−0.917***	0.000	−0.891***	0.000	−0.826***	0.000	−0.868***	0.000
Long-run effects										
CGI	−0.678***	0.000	−0.338***	0.000	−0.245***	0.000	−0.104***	0.000	−0.097***	0.000
EGY	0.741***	0.000	0.312***	0.000	0.227***	0.000	0.113***	0.008	0.100***	0.000
EG	0.213***	0.000	−0.016***	0.000	0.039***	0.000	0.124***	0.000	0.201***	0.000
FDI	−0.398**	0.047	−0.775***	0.000	−0.516***	0.000	−0.356**	0.017	−0.804***	0.000
HDI	−0.615***	0.000	−0.780**	0.033	−0.604***	0.000	−0.219***	0.000	−0.199***	0.000
PGR	0.154***	0.000	0.274*	0.084	0.418***	0.000	0.309***	0.000	0.113***	0.000
TOP	0.085	0.837	0.068	0.396	0.047***	0.000	0.033***	0.000	0.010***	0.000
URB	0.467***	0.000	0.853***	0.009	0.569***	0.000	0.266***	0.000	0.094***	0.000
Diagnostic checks										
Normality test	1.19	0.310	0.87	0.525	1.07	0.378	1.65	0.275	1.32	0.398
F-statistics	4.42***	0.000	4.38***	0.000	5.13***	0.000	3.55***	0.000	4.83***	0.000
CD-statistics	1.83	0.445	1.14	0.476	0.994	0.510	0.15	0.880	0.65	0.517
Adjusted R-squared	0.47		0.46		0.52		0.42		0.81	
Observations	3960		1518		1034		814		615	
Groups	180		69		47		37		28	
Abs/group	22		22		22		22		22	

***, **, and * indicate significance at 1%, 5%, and 10%, respectively. Observations are adjusted after lag of the dependent variables.

For the CGI-CO_2_ nexus—the central theme of the study—the results indicate that, though the sign of the coefficient is as expected, the short-run effects are insignificant across all panels, while the long-run estimates are significant at a 1% level. We find that a 1 percent increase in CGI causes CO_2_ emissions to decrease by 0.678 metric tons per capita in the full panel, all other things being equal. When we disaggregate it by income groupings, the results show that a 1% increase in CGI can reduce CO_2_ emissions by 0.338, 0.245, 0.104, and 0.097 metric tons per capita in the high-, upper middle-, lower middle-, and low-income panels, respectively. Intuitively, the results display two key findings. First, it demonstrates that good governance is an essential tool to reduce CO_2_ emissions. It is more about institutional governance and sound administrative settings for effectively channeling the available resources toward sustainable environmental quality. Second, keeping the first implication in place, the results also indicate that the size of the effects of CGI substantially differs across income-level groups. It indicates that high-income countries enjoy more favorable effects generated through good governance to reduce environmental degradation, while it keeps reducing in upper-middle-income countries and even lowers in lower-middle-income and low-income countries. This might be due to the financial, technical, and social commitment of the countries towards the implementation of good governance. The findings are theoretically valid and acceptable. High-income economies, comparatively, have institutionalized good governance as an integral part of their normal administrative endeavors, while a high corruption tendency and lower rule of law are signaled in low-income economies. Our results are partially consistent with the findings of Vogel^89^, Bhattarai and Hammig^90^, Ehrhardt-Martinez et al.^91^, Cole and Neumayer^92^, Welsch^93^, Esty and Porter^94^, Fan et al.^95^, Culas^96^, Newell^97^, Berkman and Young^98^, Bulkeley^99^, Arvin and Lew^100^, Pour^101^, Newell et al.^102^, and Jahanger et al.^41^, who employed a single dimension and found that governance (institutional quality) has a negative impact on CO_2_ emissions. Nevertheless, our findings fully support the outcome of studies conducted by Shabir et al.^38^, Wang et al.^39^, Sibanda et al.^28^, and Xaisongkham and Liu^40^.

In terms of the control variables, the findings show that EGY has a significant impact on CO_2_ emissions both in the short- and long-run across all panels. It indicates that in the short (long) run, a unit increase in EGY causes CO_2_ to increase by 0.328 (0.741), 0.717 (0.312), 0.522 (0.227), 0.191 (113), and 0.229 (0.100) metric tons per capita in the full, HIC, UMIC, LMIC, and LIC panels, respectively. The results imply that higher EGY produces more CO_2_ emissions. These findings show that EGY is yet another vital component that directly leads to the degradation of environmental quality worldwide. Due to rapid development, energy demand has been increasing around the world^103^. The burning of fossil fuels is used to meet a sizable percentage of this need. As a result, energy use significantly contributes to the decrease in environmental quality. Our results support the findings of Javid and Sharif^104^ for Pakistan; Shahbaz et al.^105^ for low, middle, and high-income countries; Farhani and Ozturk^106^ for Tunisia; Beşe and Kalayci^107^ for Egypt, Kenya, and Turkey; and Adebayo and Kirikkaleli^108^ for Japan; but contrast with those of Jebli et al.^109^ for OECD member countries; and Shafiei and Salim^110^, who, respectively, provided significant statistical evidence that more EGY has a reversible effect on CO_2_ emissions.

Except for the high-income panel, the coefficient of EG yields a positive sign at 10% significance, implying that EG accelerates CO_2_ emissions in UMIC, LMIC, LIC, and the full panel. Specifically, a 1% increase in EG causes CO_2_ emissions to increase by 0.021 (0.213), 0.047 (0.039), 0.061 (0.124), and 0.023 (0.201) metric tons per capita in the short (long) run, respectively, in the full, upper-middle-income, lower-middle-income, and low-income panels. These results correspond to those of Pilatowska et al.^111^ for the EU, Kasman and Duman^112^ for new EU members, Bekun et al.^113^ for 16 EU members, Saidi and Rahman^114^ for OPEC countries, and Khan^115^ for South Asian economies. The results are linked to stylized facts. It is expected that environmental quality will pay a price with an increase in overall economic output and national consumption. This implies that when the use of non-renewable resources increases, environmental degradation also increases, and thus, the potential loss of environmental ecosystems is only one of the negative effects of rapid economic growth on the environment. However, not all types of growth harm the environment. A sound allocation of funds to environmental preservation when real earnings rise is found to be effective and, as such, good governance.

Altogether, financial development, as proxied by the financial development index (FDI), negatively affects CO_2_ emissions. Literally, it was expected that a well-developed financial sector would facilitate enhanced access to higher investments in lower carbon emission production that significantly decreased CO_2_. Magazzino^116^ also found that financial development has a negative impact on CO_2_ emissions. Further studies by Al-Mulali et al.^117^, Tang and Tan^118^, Ho and Ho^119^, and Rahman and Alam^20^ also emphasize that a well-developed financial sector and access to credit significantly reduce CO_2_ emissions due to informed and well-thought-out investments in low-carbon-producing projects. In the purview of human interaction with the environment, we regressed HDI on CO_2_ emissions and found that, in contrast to a vast number of prior studies, HDI is significant for reducing CO_2_ emissions in the long run. This might be due to the selection of proxies. Before augmenting HDI, we regressed HCI (human capital index) and found that it has a rather positive impact on CO_2_ emissions. While HCI does not fully cover all aspects of human interaction with society, we swapped it with HDI. Our results align with Bano et al.^68^, Çakar et al.^120^, Zhu^121^, and Song et al.^122^, who also found that human capital development is a crucial predictor of maintaining a low-carbon environment. Moreover, the findings also indicate that PGR is strongly significant in impacting CO_2_ emissions across all panels. It shows that a 1% increase in PGR causes CO_2_ emissions to increase by 0.159, 0.19, 0.379, 0.301, and 0.121 metric tons per capita in the full, high, upper-middle, lower-middle, and low-income panels, respectively, in the long run, while short-run effects are insignificant. The positivity of PGR can be traced through two conduits. First, growth in the population, especially uncontrolled growth, increases the demand for energy consumption, industry, and transportation alike, which significantly contributes to increasing CO_2_. Second, PGR is a significant predictor of increases in greenhouse gas emissions. Studies by Dong et al.^123^, Weber and Sciubba^23^, and Ray and Ray^124^ support our findings on the positive impact of PGR on CO_2_ emissions.

With respect to urbanization (URB), the findings reveal that while its short-run effects are only evident in the full and low-income panels, its long-run effects are significant across all panels. It shows that URB is another factor that, without exception, increases CO_2_ emissions. The findings are linked to the fact that higher urbanization results in greater deforestation, higher freshwater extraction, and the utilization of more carbon-producing goods that reduce environmental quality in the long run^125^. Prior studies by Akalin et al.^126^, Nathaniel^127^, Kahouli et al.^128^, and Radoine et al.^129^ also support our findings. Finally, our findings with respect to trade openness (TOP) are somehow similar to the existing literature. We only find that TOP is significant in high-income panels in the short run, while it only affects CO2 emissions in UMIC, LMIC, and LIC panels in the long run. Overall, our findings indicate that TOP would facilitate higher CO2 emissions. Studies that concur with our findings include Ertugrul et al.^130^, Ragoubi and Mighri^131^, Dou et al.^22^, Chen et al.^132^, and Adebayo et al.^108^, though studies by Mahmood et al.^133^ and Yu et al.^134^ found negative and spillover effects of TOP on CO_2_ emissions, respectively.

All results reported in Table 7 are statistically robust. Diagnostic checks are reported underneath every panel estimation of the CS-ARDL model. They report two important facts. First, CD is corrected across all panels. Second, residuals are normally distributed and hold the underlying assumptions. Furthermore, to confirm the robustness of our estimates, we employed the FMOLS and DOLS methods and reported their results in Table 8. Though the estimated long-run effects obtained from FMOLS and DOLS are slightly different than those obtained from the CS-ARDL model, they hold identical signs. Similar methods of robustness testing are common in the existing literature^38,103^.

**Table 8:** FMOLS and DOLS test results.

Models estimated	(1) Full panel		(2) HIC		(3) UMIC		(4) LMIC		(5) LIC	
	Statistics	p-value	Statistics	p-value	Statistics	p-value	Statistics	p-value	Statistics	p-value
FMOLS										
CGI	−0.705**	0.012	−0.325***	0.000	−0.236***	0.000	−0.198***	0.000	−0.101***	0.000
EGY	0.696**	0.044	0.370***	0.000	0.214**	0.042	0.106***	0.000	0.099***	0.000
EG	0.225***	0.000	0.017***	0.000	−0.035***	0.000	−0.145***	0.000	0.213***	0.000
FDI	−0.401***	0.000	−0.790***	0.000	−0.499***	0.000	−0.314***	0.000	−0.791***	0.000
HDI	−0.599*	0.068	−0.588***	0.000	−0.617***	0.000	−0.211***	0.000	−0.206***	0.000
PGR	0.161***	0.000	0.140**	0.035	0.405***	0.008	0.287***	0.000	0.118***	0.000
TOP	0.091***	0.000	0.020*	0.091	0.044*	0.072	0.029***	0.000	0.017***	0.000
URB	0.471***	0.000	0.490***	0.000	0.558**	0.011	0.253***	0.000	0.101***	0.000
Diagnistic checks										
R-squared	0.618		0.889		0.710		0.816		0.888	
Normality test	1.45	0.225	0.313	0.450	1.32	0.275	1.67	0.205	1.45	0.256
Observations	3420		1342		893		703		532	
DOLS										
CGI	−0.712***	0.009	−0.360***	0.000	−0.241***	0.000	−0.201***	0.000	−0.099***	0.000
EGY	0.683**	0.038	0.318***	0.000	0.196***	0.000	0.111***	0.000	0.104***	0.000
EG	0.219***	0.000	0.015*	0.051	−0.033*	0.081	−0.137**	0.045	0.189***	0.000
FDI	−0.413***	0.000	−0.571***	0.000	−0.510***	0.000	−0.372***	0.000	−0.815***	0.000
HDI	−0.606**	0.048	−0.592***	0.000	−0.658***	0.000	−0.235***	0.000	−0.192***	0.000
PGR	0.159***	0.000	0.129*	0.076	0.379*	0.061	0.301***	0.000	0.121***	0.000
TOP	0.088***	0.000	0.029***	0.000	0.046***	0.000	0.025***	0.000	0.012***	0.000
URB	0.463***	0.000	0.505***	0.000	0.551***	0.000	0.267***	0.000	0.091***	0.000
Diagnistic checks										
R-squared	0.778		0.928		0.814		0.883		0.865	
Normality test	1.670	0.110	0.930	0.303	1.69	0.109	0.74	0.310	1.33	0.320
Observations	3420		1342		893		703		532	

***, **, and * indicate significance at 1%, 5%, and 10%, respectively.

## Conclusions

Environmental degradation represents a serious concern for everyone. Governments constantly try to find rational remedies to minimize the effects of environmental degradation. However, there are various factors that contribute to environmental degradation, of which CO_2_ is the most important. To that end, this study investigates the effects of good governance, energy consumption, economic growth, financial development, and other socioeconomic predictors on CO_2_ in a large panel consisting of 180 countries classified as full, high-income, upper-middle-income, lower-middle-income, and low-income groups over the period from 1999 to 2021. Observing stationarity properties and panel heterogeneity, the study utilized the CS-ARDL model, which was statistically plausible to account for the rejected null of no cross-sectional dependence in the panels. Moreover, to capture the comprehensive effects of good governance, the key variable of interest, the study constructed a composite governance index (CGI) using six indicators of governance under the accountability, participation, and predictability dimensions.

For the full panel (say, the global panel) and all income-level groups, the findings suggest that there exists a long-run relationship between CGI, CO_2_ emissions, and other control variables. The results obtained from the CS-ARDL technique indicate that CGI has only long-run effects on CO_2_ emissions across all panels; short-run effects were found to be insignificant. However, we found that CGI is an important factor contributing to reducing global CO_2_ emissions and improving environmental quality, but the magnitude of its contribution differs according to the economic size and presentation of the underlying countries. Additionally, economic growth was found to have a negative impact on CO_2_ emissions in the high-income panel, while it exerts positive effects on the subject in the full, upper-middle-income, lower-middle-income, and low-income groups. Similarly, energy consumption, with no exceptions, was found to have a significant role in environmental degradation worldwide. Furthermore, the results demonstrate that trade openness can only be harmful to environmental quality in the upper-middle-income, lower-middle-income, and low-income groups; full panel and high-income countries were found to be effectless. While the findings reveal that population growth and urbanization directly contribute to environmental deterioration, financial development has favorable effects on improving the quality of the environment worldwide. It implies that as a result of population growth and higher urbanization, demand for energy, industry, and transportation increases, resulting in increased CO_2_ emissions. Comparatively, the results demonstrate that financial development negatively effects CO_2_, implying that a well-developed financial sector facilitates enhanced access to higher investments in lower carbon production that significantly decreases CO_2_ emissions.

### Policy implications

Our findings highlight several policy implications that are specifically discussed as follows:

i. Altogether, good governance is crucial to maintaining and improving global environmental quality, regardless of the size of its effects. It is imperative to institutionalize good governance to encourage efficient reiteration and utilization of resources for higher environmental preservation.
ii. For high-income countries, economic growth is no longer a silver bullet to recast environmental quality; rather, it is regarded as another essential tool for upper-middle-income, lower-middle-income, and low-income countries to reverse the negative impact of rapid growth on environmental quality. This suggests specific policy reorientations in their growth-targeting regimes.
iii. Although budget implications are real concerns in low-income countries, the findings suggest that efficient energy consumption and the deployment of innovative ecological technologies in the production sector of the economy can spur environmental quality.
iv. All in all, the growth and massification of populations in urban areas are harmful to environmental quality. Specific policy adjustments are required to facilitate the economic shift and ensure an even population distribution.
v. With no exception, human interaction with the environment is also a determinantal factor. Well-thought-out investments in human capital development can result in increased education and awareness to preserve environmental quality.
vi. From a global perspective, while many factors contribute to global warming, CO_2_ is the most important, implying economies must follow global policy incentives and implement new mechanisms to reduce CO_2_, such as better forest management, taxes on ecologically harmful behaviors, increasing the total cumulative area of the Earth sheltered in forests, and smoothing the transition to electric and hybrid automobiles.

### Limitations

This study highlights an important promotional role for the links between good governance and a sustainable environment and has provided a comprehensive statistical scenario of the effects of good governance on environmental quality from a global perspective, but it suffers from one major limitation: the exclusion of armed conflict effects from the analysis in some of the countries due to the unavailability of relevant datasets. Future studies can overcome this empirical shortcoming, depending on the availability of the required data.

### Supplementary Information

Supplementary Information.

## Data Availability

The datasets relevant to governance indicators come from WGI, FDI is collected from IMF, HDI has been compiled from PWT 9.0 (Penn World Table), sourced from Feenstra et al. (2015). The data for all other variables have been collected from World Development Indicators (WDI). All datasets are freely available and accessible to the public.
