# Supplementary material for: Linking governance with environmental quality: a global perspective
Source: Sci Rep. 2023 Sep 12;13:15086. doi: 10.1038/s41598-023-42221-y (PMC10497530; doi:10.1038/s41598-023-42221-y)
Supplement: Supplementary file 1 — Supplementary Information. [file 41598_2023_42221_MOESM1_ESM.docx]

**Appendix A: CGI Construction**

**A1 Accountability dimension**

The CGI focuses on three key dimensions, such as accountability, participation, and transparency (say, predictability), that are globally accepted as the elements of good governance (Barro, 1991; Barro and Sala, 1992; Hulten, 1996). The accountability dimension reflects the extent to which a state is responsible for providing answers for its actions. The control of corruption and the rule of law are the indicators used for this dimension. We first assigned weights to each indicator using the descriptive statistics. Table A1 provides detail on the allocated weights to each dimension for the full and income-level panels.

**A2 Transparency dimension**

For transparency dimension, regulatory quality and government effectiveness are used as appropriate indicators (Montes et al., 2019). This dimension measures the extent to which a government is transparent with respect to the development and implementation of regulations and its effectiveness in providing transparent and discrimination-free services to people.

**A3 Participation dimension**

For the participation dimension, two governance indicators, such as political stability and voice and accountability, are used as appropriate measures. This dimension is used to measure the extent to which the government is effective in terms of citizens’ participation in electing their government of choice, freedom of association, free media, the undue participation of people in politics, and political stability.

**Table A1** CGI aggregate index—weight of indicators.

| Dimension | Governance indicators | Allocated weights | | | | |
| --- | --- | --- | --- | --- | --- | --- |
|  |  | Full panel | HIC | UMIC | LMIC | LIC |
| Accountability | [1]. Control of corruption  [2]. Rule of law | 0.90  0.80 | 0.75  0.77 | 0.42  0.43 | 0.29  0.29 | 0.19  0.17 |
| Transparency | [1]. Regulatory quality  [2]. Government effectiveness | 0.80  0.80 | 0.77  0.77 | 0.45  0.45 | 0.28  0.29 | 0.17  0.14 |
| Participation | [1]. Vice and accountability  [2]. Political stability | 0.85  0.80 | 0.77  0.67 | 0.49  0.45 | 0.32  0.31 | 0.20  0.20 |

**A4 Construction of dimensions**

Following Sarma (2012), for the construction of dimensions, the study employs empirical minimum and maximum observations for specific governance indicators. To that end, the first step is to calculate the relevant indicators for each dimension—that is, the accountability, transparency, and participation (predictability)—using the following equation:

$d_{i}=\varpi_{i}\frac{A_{ij,t}-\delta_{i}}{\Delta_{i}-\delta_{i}},$ (A1)

where $d_{i}$ is the value of the relevant governance indicator for the $ith$ dimension, $\varpi_{i}$ presents the weight of the governance indicator for the $ith$ dimension, $A_{i}$ is the actual value of the specific governance indicator for the panel $j$ in time $t$, $\Delta_{i} \text{and} \delta_{i}$ are the upper and lower limits of the indicators, respectively, for the $ith$ dimension. Equation (A1) computes the $d_{i}$, which is the normalized score of the specific governance indicator for relevant good governance dimension. The higher the value of $d_{i}$, the higher the governance practice in the particular indicator will be. Next, the study calculates the normalized Euclidian distance among $(X)$ and worst $(O)$positions and for the normalized inverse Euclidian distance among $(X)$ and the ideal $(I)$on the $nth$ space of the dimensions by the following equations:

$X_{1}=\frac{\sqrt{d_{1}^{2}+d_{2}^{2}+d_{3}^{2}+...+d_{n}^{2}}}{\sqrt{I_{1}^{2}+I_{2}^{2}+I_{3}^{2}+...I_{n}^{2}}},$ (A2)

$X_{2}=\frac{\sqrt{\left(\varpi_{1}-d_{1}\right)^{2}+...+\left(\varpi_{1}-d_{n}\right)^{2}}}{\sqrt{\left(I_{1}^{2}+I_{2}^{2}+I_{3}^{2}+...+I_{n}^{2}\right)}},$ (A3)

Equations (A2) and (A3) is based on the idea of distance points shown $d_{1},d_{2},...,d_{n}$, from the worst $(O)$and an ideal position $I_{1},I_{2},...,I_{n}$ (Omar and Inaba, 2020). Finally, the composite governance index with multiple dimensions can be constructed using the following equation:

$CGI_{i}=\frac{1}{2}\left(X_{1}+X_{2}\right)$ (A4)

where CGI is the composite governance index, presenting values from 0 (imperfect) to 1 (perfect) governance. However, this methodology has been recently used by Swamy (Park and Mercado, 2015; Salazar-Cantú et al., 2015; Swamy, 2014) for constructing a composite financial inclusion index, but innovatively, for the first time, the present study attempts to classify the WGI indicators under three dimensions and construct a composite governance index—that is, the CGI—to test its effects on CO_2_ emissions.
